# Comparing and combining xevinapant with ATR and PARP inhibition for the radiosensitization of HPV-negative HNSCC cells

**DOI:** 10.1038/s41598-026-38550-3

**Published:** 2026-02-11

**Authors:** Julius Roehrle, Adriana Perugachi-Heinsohn, Fruzsina Gatzemeier, Sabrina Christiansen, Cordula Petersen, Kai Rothkamm, Christian Betz, Malte Kriegs, Henrike Barbara Zech, Thorsten Rieckmann

**Affiliations:** 1 Department of Otorhinolaryngology, University Medical Center Hamburg-Eppendorf, Martinistrasse 52, 20246 Hamburg, Germany; 2 Department of Radiation Oncology, University Medical Center Hamburg-Eppendorf, Martinistrasse 52, 20246 Hamburg, Germany

**Keywords:** Head and neck cancer, Molecular targeting, Radiosensitization, Programmed cell death, SMAC, PARP, ATR, Cancer, Oncology

## Abstract

Radiochemotherapy with the apoptosis stimulator xevinapant demonstrated superiority over radiochemotherapy in a randomized phase 2 trial in head and neck squamous cell carcinoma (HNSCC) but the subsequent phase 3 trial failed. Here, we compared the radiosensitization through xevinapant with two other emerging approaches, the inhibition of ATR and PARP through tuvusertib and olaparib, in a panel of four radioresistant HPV-negative HNSCC cell lines (HSC4, UT-SCC-60A, SAS, SAT). Without irradiation the analyses of proliferation and colony formation showed varying sensitivities of the cell lines towards the different agents and their combinations. When combined with radiation in colony formation assays, we observed moderate radiosensitization through xevinapant in individual cell lines but not in general. Both tuvusertib and olaparib induced stronger radiosensitization than xevinapant and combining both agents resulted in especially profound radiosensitization in three out of the four cell lines tested, whereas their combination with xevinapant or the combination of xevinapant with cisplatin was less effective. Assessment of cell death induction via annexin V/DAPI staining failed to generally predict cytotoxicity or radiosensitization of these approaches. Overall, our data are in line with the recent failure of the phase 3 TrilynX trial and suggest further investigation of ATR and PARP inhibition for the curative treatment of HPV-negative HNSCC.

**Supplementary Information:**

The online version contains supplementary material available at 10.1038/s41598-026-38550-3.

## Introduction

The standard therapeutic approach for locally advanced head and neck squamous cell carcinoma (HNSCC) typically involves either definitive cisplatin-based chemoradiotherapy or primary surgical resection followed by adjuvant (chemo)radiotherapy. However, treatment-associated morbidity remains substantial, owing to both acute and long-term, partly irreversible side effects, including severe mucositis, dysphagia, xerostomia, hearing impairment, and nephrotoxicity. In patients with human papillomavirus (HPV)-negative HNSCC, treatment outcomes remain suboptimal due to frequent resistance to radio- and chemotherapy and a substantial fraction of elderly or fragile patients is not eligible for standard cisplatin-based chemoradiotherapy^1–3^. Molecular targeting strategies aiming at enhancing tumor radiosensitivity offer a potential alternative to concurrent chemotherapy. So far, the anti-EGFR monoclonal antibody cetuximab is the only approved substance in this setting but considerable doubts exist regarding its efficacy when combined with radiation^4^.

Apart from EGFR inhibition, various other approaches for radiosensitization exist. One is induction of cell death by pro-apoptotic substances. The SMAC mimetic xevinapant (Debio-1143) inhibits inhibitor of apoptosis proteins (IAPs), was reported to radiosensitize HPV-negative HNSCC cell lines^5^ and its addition to radiochemotherapy is the only approach to ever demonstrate superiority over primary concurrent radiochemotherapy in a randomized clinical trial in HNSCC^6^. However, the phase 3 TrilynX trial following the successful phase 2 trial was stopped after a planned interim analysis. A recent analysis unexpectedly even demonstrated inferiority of the xevinapant arm with increased toxicity and reduced survival^7^. All other open and planned trials with xevinapant in HNSCC have been cancelled.

Other emerging approaches for potentially tumor-specific radiosensitization include inhibition of the DNA damage response and DNA repair pathways, such as the use of PARP inhibition or inhibitors of the ATR/Chk1/Wee1 axis^8,9^. The PARP inhibitor olaparib (AZD-2281) is already approved without irradiation in ovarian, breast, prostate and pancreatic cancer, mostly in the context of homologous recombination deficiency through BRCA mutations. The inhibition of the ATR/Chk1/Wee1 axis had first been focused on Chk1 and Wee1 inhibitors but the development of many substances, such as prexasertib and adavosertib, has been largely discontinued by the respective companies. Instead, ATR inhibition is currently considered as a less toxic and effective option and is the clinical focus with and without irradiation and when combined with other substances^10,11^.

Here we have compared the radiosensitizing potential of the cell death stimulator xevinapant to the ATR inhibitor tuvusertib (M1774) and the PARP inhibitor olaparib and tested whether a combination of these agents may result in enhanced effectiveness.

## Results

### Dose finding for tuvusertib

**Fig. 1:**
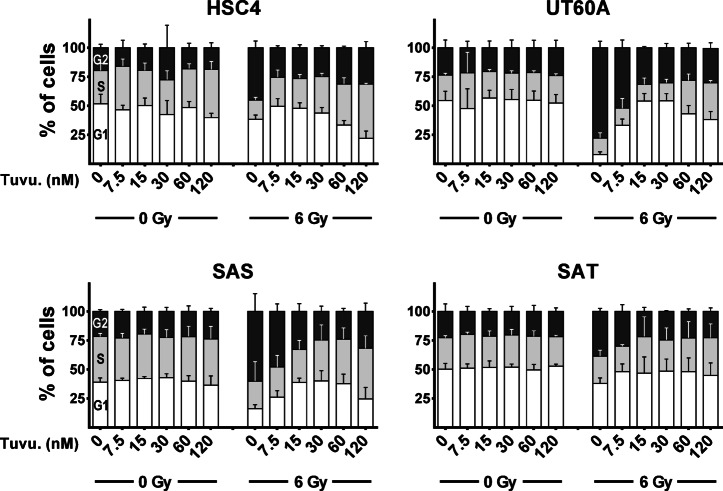
Radiation induced G2 arrest. Exponentially growing cells were treated with increasing concentrations of tuvusertib and after 2 h irradiated as indicated. At 12 h after irradiation the cells were fixed, and the cell cycle distribution assessed by DAPI staining and flow cytometry. Results are based on 3 individual experiments per cell line. Graphs display mean and standard deviation.

While xevinapant and olaparib have been used in various clinical and preclinical studies, especially preclinical data for the ATR inhibitor tuvusertib are sparse^12^ and no data for the combination with irradiation are available. ATR is well known to be required for the radiation-induced arrest in the G2 cell cycle phase, and we first assessed the ability of different concentrations of tuvusertib to inhibit this arrest in HPV-negative HNSCC cell lines (Fig. 1). A low dose of 30 nM was sufficient to revert radiation-induced G2 arrest at 12 h after irradiation in all four cell lines tested and was used in the following experiments while xevinapant and olaparib were both used at clinically achievable concentrations of 1 µM^13–15^.

### Inhibition of cell proliferation

**Fig. 2:**
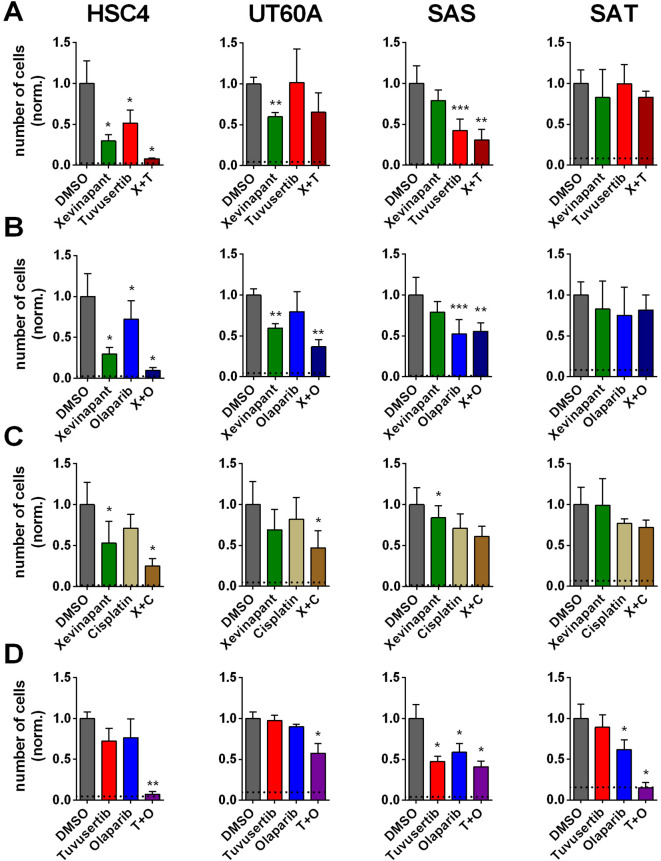
Cell proliferation. Cells were seeded and after 3 h treated with substances as indicated. Five days later the respective numbers of cells were assessed. (**A**) Treatment with xevinapant and tuvusertib, (**B**) treatment with xevinapant and olaparib, (**C**) treatment with xevinapant and cisplatin and **(D)** treatment with tuvusertib and olaparib. Graphs (**A**) and (**B**) share identical DMSO and xevinapant values because the results were generated in combined experiments. Data are presented as normalized to the mean of the respective DMSO controls. Significant differences to DMSO-treated samples are indicated with *, ** and *** indicating p < 0.05, p < 0.01 and p < 0.001, respectively (paired, two-tailed Student’s t-test). Dotted lines indicate the number of cells seeded (normalized). Results are based on at least 3 individual experiments per cell line. Graphs display mean and standard deviation.

As a first comparative analysis of the effect of the three radiosensitizers, we assessed their influence on tumor cell proliferation. As single agents, we mostly observed moderate inhibitory effects and partly stronger inhibition, for example for tuvusertib in SAS or xevinapant in HSC4 cells. In any case, the cells were still proliferating (Fig. 2). When substances were combined, we observed profound growth inhibition in HSC4 cells, suggestive of synergistic effects. However, such effects were cell-line specific, as they were not observed over the whole cell-line panel for any of the inhibitor combinations and also not for the combination of xevinapant with a moderate dose of 250 nM cisplatin.

### Induction of cell death

**Fig. 3:**
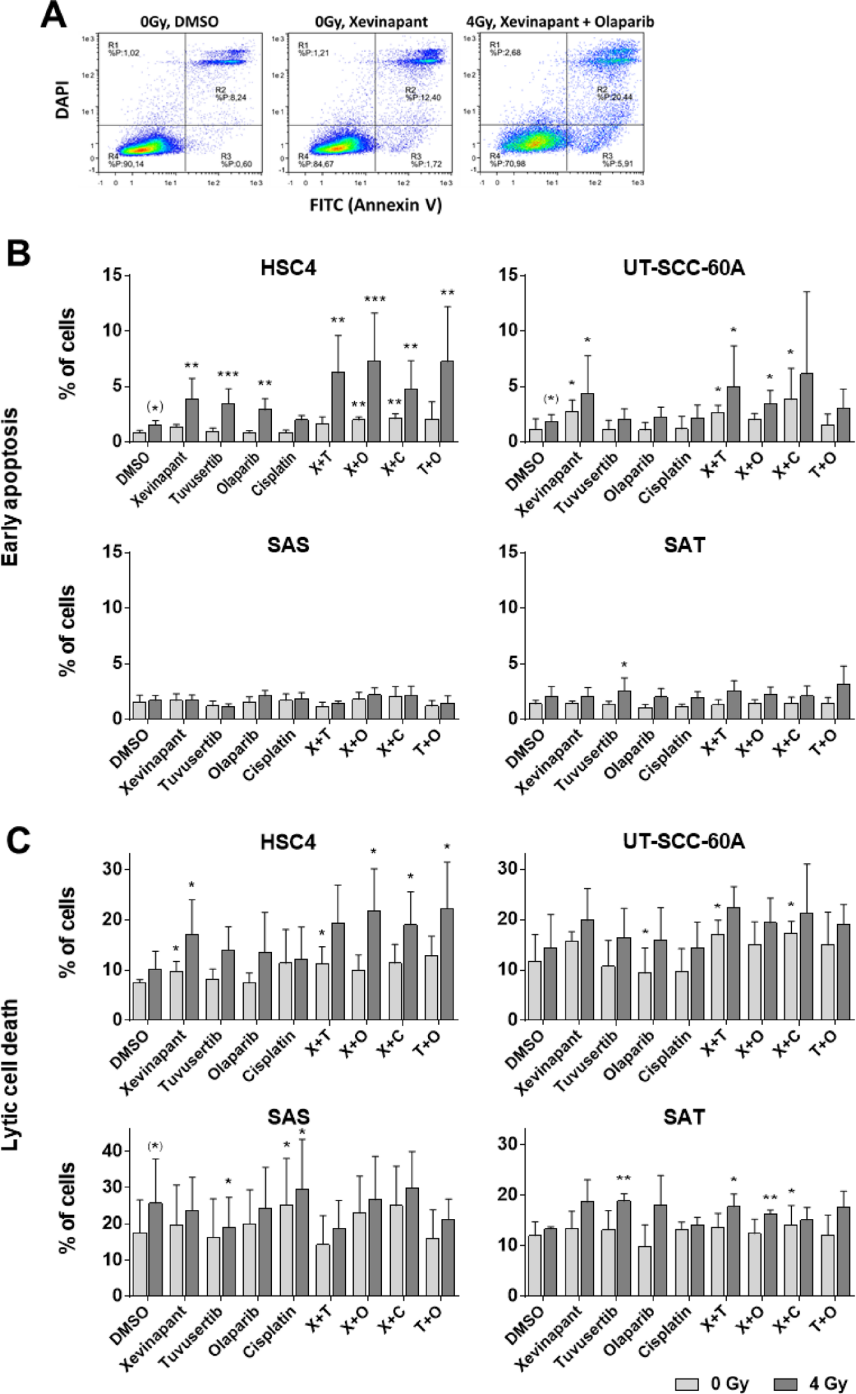
Cell death induction. Cells were seeded, after 3 h treated with substances and after an additional 2 h irradiated as indicated. 48 h later cells were harvested, stained for annexin V and DAPI and analyzed using flow cytometry. (**A**) Examples and gating. Events in the lower right rectangle were counted as early apoptosis and in the upper right rectangle as lytic cell death. Shown are HSC4 cells. (**B**) Quantification of early apoptosis and (**C**) quantification of lytic cell death. Significant differences to the respective irradiated or non-irradiated DMSO control are indicated with *, ** and *** indicating p < 0.05, p < 0.01 and p < 0.001, respectively (paired, two-tailed Student’s t-test). Asterisks in brackets indicate significant differences between irradiated and non-irradiated DMSO controls. Results are based on at least 4 individual experiments per cell line. Graphs display mean and standard deviation.

SMAC mimetics are apoptosis- and partly necroptosis-inducing agents^16,17^. We therefore tested to what extent single and combined treatment with or without ionizing irradiation induced early apoptosis or lytic cell death using combined annexin V and DAPI staining (Fig. 3A). In general, the fractions of lytic cells were clearly higher than the fractions of cells in early apoptosis. Regarding early apoptosis, xevinapant resulted in a moderate increase upon irradiation in HSC4 cells and with and without irradiation in UT-SCC-60A. Particularly in HSC4, xevinapant, olaparib, and tuvusertib all significantly augmented the radiation-induced increase in early apoptosis and the effect was most pronounced upon combined inhibition. However, in SAS and SAT cells, none of the applied treatments induced any profound increase in the number of early apoptotic cells (Fig. 3B). Regarding lytic cell death, all cell lines demonstrated higher background values as compared to early apoptosis. Treatment without irradiation in general induced only moderate effects. After irradiation, significantly enhanced lytic death was again most frequently increased in HSC4 cells, under xevinapant as well as combined treatment (Fig. 3C).

Overall, cell death analyses suggest that mostly HSC4 may be radiosensitized when combining xevinapant with one of the other inhibitors or cisplatin but do not suggest a profound general increase in radiation sensitivity. Analyzing overall cell death (early apoptosis + lytic cell death) confirmed this notion (Supplementary Fig. 1).

### Clonogenic survival

Xevinapant had previously been reported to radiosensitize five out of a panel of six HNSCC cell lines, including FaDu cells. Sensitization required prolonged exposure to the drug after irradiation, and often did not show a clear concentration dependence^5^. We therefore first tested concentrations of 0.25, 1 and 4 µM in FaDu cells in colony formation assays as the gold standard for radiation sensitivity assessment. We also observed highly similar radiosensitization with the different concentrations, albeit at a more modest level, confirming that our clinically relevant dose of 1 µM is suitable (Supplementary Fig. 2).

**Fig. 4:**
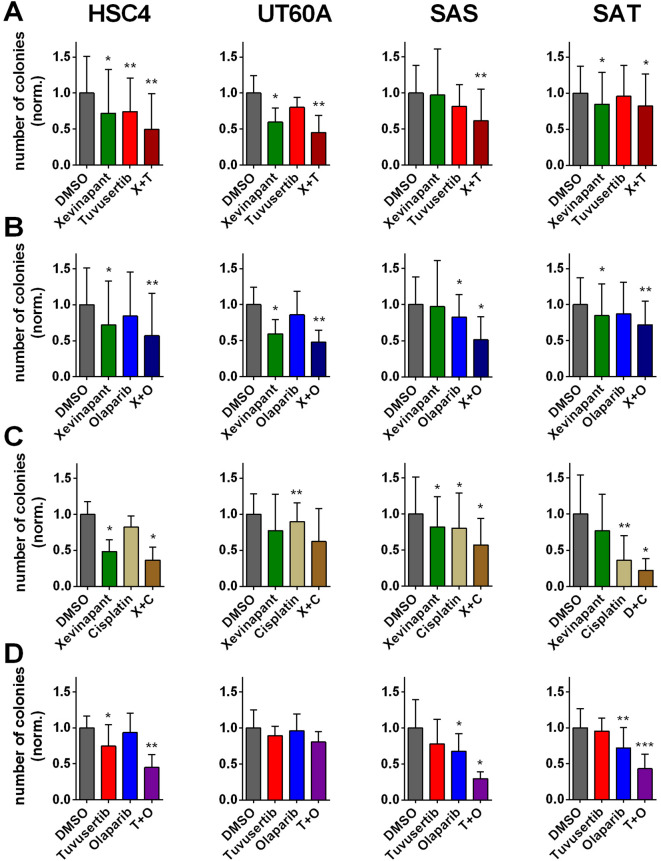
Colony formation. Cells were seeded in defined low numbers and after 3 h treated with substances as indicated for 26 h, except for xevinapant with an incubation time of 1 week. Cultures were incubated until formation of colonies. (**A**) Treatment with xevinapant and tuvusertib, (**B**) treatment with xevinapant and olaparib, (**C**) treatment with xevinapant and cisplatin and (**D**) treatment with tuvusertib and olaparib. Graphs (**A**) and (**B**) share identical DMSO and xevinapant values because the results were generated in combined experiments. Significant differences to the respective solvent controls are indicated with *, ** and *** indicating p < 0.05, p < 0.01 and p < 0.001, respectively (paired, two-tailed Student’s t-test). Results are based on at least 4 individual experiments per cell line. Graphs display mean and standard deviation.

Next, to finally compare the effects of single and combined inhibition on cell survival, we conducted colony formation assays in our radioresistant^18,19^ HPV-negative cell panel. First, we compared plating efficiencies of the non-irradiated samples as a means of in vitro cytotoxicity. Responses to the inhibitors were overall similar to those in the proliferation assays but with partly lower extent of inhibition and fewer differences between the cell lines. This may be explained by only transient treatment in colony formation assays and the ability to grow to colonies after substance withdrawal after either 26 h or, in case of xevinapant, after 1 week. Combined inhibition was often more effective than single inhibition but was not generally suggestive of more than additive effects (Fig. 4).

### Radiosensitization

**Fig. 5:**
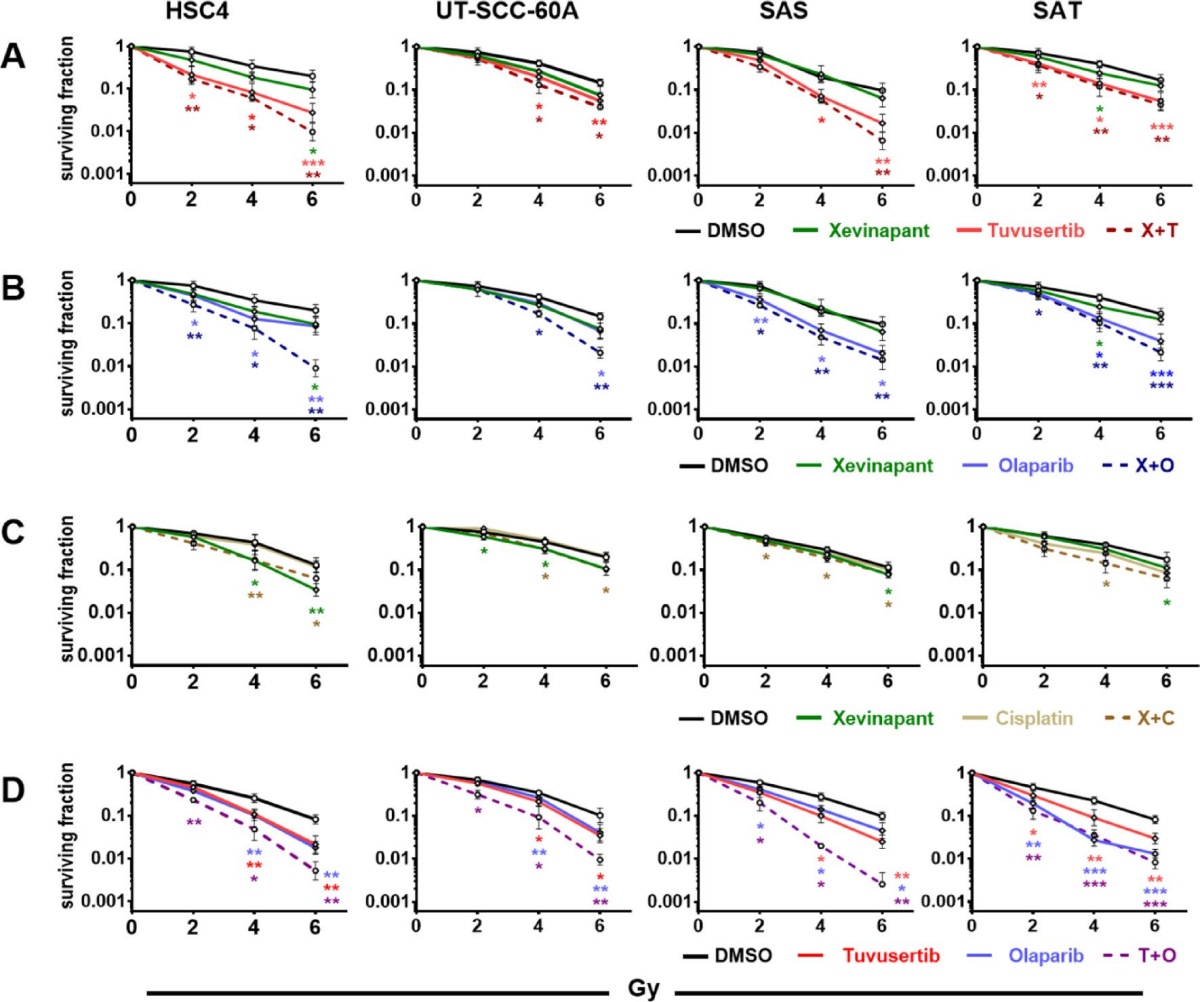
Radiosensitization. Cells were seeded in defined low numbers and after 3 h treated with substances as indicated for 26 h, except for xevinapant with an incubation time of 1 week. Cultures were incubated until formation of colonies. Graphs represent the normalized dose response curves from the same experiments as shown in Fig. 4. (**A**) Treatment with xevinapant and tuvusertib, (**B**) treatment with xevinapant and olaparib, (**C**) treatment with xevinapant and cisplatin and (**D**) treatment with tuvusertib and olaparib. Graphs (**A**) and (**B**) share identical DMSO and xevinapant values because the results were generated in combined experiments. Significant differences to the respective solvent controls are indicated with *, ** and *** indicating p < 0.05, p < 0.01 and p < 0.001, respectively (paired, two-tailed Student’s t-test). Results are based on at least 4 individual experiments per cell line. Dose–response curves display mean and standard deviation.

In terms of radiosensitization, we observed overall lower effectiveness of xevinapant compared with tuvusertib and olaparib, which induced radiosensitization in all four cell lines tested. Xevinapant demonstrated no or only minimal effectiveness in two strains (SAT and SAS) (Fig. 5A–D). The combination of xevinapant and cisplatin, as used in the conflicting clinical studies mentioned above, resulted in numerically enhanced radiosensitization in SAT, but not in any of the other strains. Cisplatin alone did not result in radiosensitization in our cell panel in line with previous data (Fig. 5C)^20^. No combination of xevinapant with another agent resulted in generally enhanced effectiveness across all cell lines. The most effective pairing was the combination with olaparib, which resulted in clearly enhanced effectiveness in HSC4 and UT-SCC-60A cells (Fig. 5B). Combining tuvusertib and olaparib resulted in clearly enhanced radiosensitization in three of the four cell lines tested (Fig. 5D).

## Discussion

The pharmacologic induction of cellular radiation sensitivity has been extensively studied with various successful in vitro approaches but little success in transferring these into the clinical setting. The apoptosis stimulating SMAC mimetic xevinapant is a rare example of a compound for which a preclinical radiosensitization was followed by a successful randomized clinical trial (phase 2)^5,6^. Unfortunately, the subsequent phase 3 TrilynX trial did not confirm the results but was stopped after an interim analysis. Recently published results even show adverse performance of the xevinapant arm with reduced overall, event-free, and progression-free survival. Locoregional control was not improved by xevinapant and distant metastases were considerably enriched. The inferior results may in part be explained by unexpected and early toxicity in the xevinapant arm that was not observed in the phase 2 study and required treatment interruptions and modifications^7^.

In this translational project initiated before the termination of the TrilynX trial, we wanted to compare the radiosensitizing potential of cell death induction through xevinapant with radiosensitization through ATR and PARP inhibition as competing approaches. We further wanted to test whether combined treatment of xevinapant with these DDR inhibitors may lead to enhanced radiosensitization with the rationale that increased residual DNA damage, replication stress or premature mitosis may result in enhanced cell death induction through xevinapant. Overall, while we confirm that xevinapant has the potential to radiosensitize HPV-negative HNSCC cells, the breadth and extent of radiosensitization in our setting was less profound than previously reported^5^. An experimental difference between our study and the study of Matzinger et al., apart from using different cell lines, is that we exchanged the media and withdrew xevinapant after one week in the colony formation assays to support the growth of colonies from surviving cells, in which xevinapant may have antiproliferative effects. A combination of xevinapant and cisplatin, which was also the setting in both randomized clinical studies mentioned above, served as a further comparison. The finding that cisplatin exerted only limited effects and no significant radiosensitization on its own may be somewhat surprising for a clinically used radiosensitizer. However, it needs to be considered that the overall survival benefit from cisplatin in HNSCC is only about 7%^21^, which likely means that only a subgroup will benefit and direct cytotoxicity on tumors and micrometastasis can also play a role. The data are further in line with previous in vitro results, where, in our hands, different HNSCC cell lines demonstrated a large variation in cisplatin sensitivity and only a fraction of cell lines was sensitized to radiation^20^. The combination of xevinapant and cisplatin enhanced radiosensitization in only one out of the four cell lines tested. With the limitation that our study used only a single moderate dose of cisplatin, the limited effectiveness of xevinapant—alone and in combination with cisplatin—is consistent with, and may partly explain, the lack of improvement in locoregional control observed in the TrilynX trial.

In a direct comparison with xevinapant, both tuvusertib and olaparib proved to be more effective radiosensitizers in HPV-negative HNSCC on the cellular level. For tuvusertib this is, to the best of our knowledge, the first report for the combination with ionizing radiation. When combining olaparib with a very moderate dose of 30 nM tuvusertib we observed a further increase in radiosensitization in three out of the four cell lines tested. Therefore, as single agents, as well as in combination, the DDR inhibition approaches in our hands are more potent than cell death induction through the SMAC mimetic xevinapant. We therefore conclude that these approaches should be further examined for their clinical potential. The combination of PARP and ATR inhibition has previously been described as a potent radiosensitization approach in glioblastoma cells^22^. Biologically similar approaches of PARP plus Wee1 or Chk1 inhibition have also resulted in profound radiosensitization in HNSCC and other cancer cell types^19,23–28^, which speaks for the robustness of the approach. An advantage of ATR as compared to Chk1 and Wee1 inhibition is its favorable toxicity profile, which has made it the most promising candidate for inhibiting the ATR/Chk1/Wee1 axis. Various approaches of combining ATR and PARP inhibition have already been made in clinical trials without irradiation and with moderate dose reductions of either one or both agents and intermittent administration schedules have demonstrated acceptable toxicity profiles^11,29,30^. Transferring radiosensitization approaches from the preclinical into the clinical setting, however, remains especially challenging. ATR inhibition is currently being tested with palliative radiation in solid tumors and in combination with curative radiotherapy in the setting of a platform study for non-small cell lung cancer but no reliable data are available so far (NCT04550104, NCT02223923). When combining olaparib with chemoradiation or with radiotherapy plus cetuximab in HNSCC the recommended dose had to be severely reduced to 25 mg once or twice daily as compared with the use of 300 mg twice daily as single agent due to acute mucosal toxicities^31,32^. So far, no data are available for the potentially less critical combination with radiotherapy alone but preliminary data from other entities also suggest a requirement for substantially reduced doses^33,34^. In glioblastoma a dose of 200 mg twice daily was recommended in a study using a reduced radiation dose of 40 Gy in patients unsuitable for radical treatment^35^. As both PARP1 and ATR serve highly important functions in the S-phase of the cell cycle^11,36^, the reason is likely a lack of highly proliferative normal tissues in the radiation field and the limited radiation intensity in the setting of that particular dose-finding study. Identifying effective and tolerable schedules therefore remains challenging as it depends on tumor location, radiation dose, and substance combinations.

Regarding the future standard curative treatment of HNSCC, pembrolizumab has recently been approved by the FDA after the positive Keynote 689 trial^37^. It was administered as neoadjuvant therapy before surgery, concomitantly with radio(chemo)therapy and as 12 cycles maintenance thereafter. This comes after a number of unsuccessful randomized trials of concomitant immune checkpoint inhibitor administration with and without maintenance, all without surgery^38–41^. We believe that this may leave opportunities for the concomitant administration of potent radiosensitizers in patients not eligible for surgery or for the concomitant administration after surgery plus neoadjuvant and maintenance pembrolizumab. Based on the in vitro radiosensitization data presented here, the inhibition of PARP and ATR as single agents or combined may represent such a highly effective alternative, for example in cisplatin-ineligible patients. Tolerable doses would need to be carefully evaluated for the inhibitors and potentially also for radiotherapy. Highly effective radiosensitization through combined treatment, for example in rapidly proliferating tumors based on Ki67 staining, may require and in principle allow a moderate reduction in radiation dose. However, translation of preclinical findings into the clinic will likely remain a major challenge in the field. In the preclinical setting, we advocate for rigorous preclinical evaluation of effectiveness in panels of cell lines and, as a second step, more physiological models. A prominent negative example is the clinical testing of cetuximab in HPV-positive HNSCC in three parallel phase 3 trials without any preceding preclinical evidence of effectiveness and with consistently negative results^42–44^. Here, preclinical studies—that were published after the trials had been initiated—suggested a complete lack of radiosensitization through cetuximab^25^ and on the contrary even radiosensitization through EGFR stimulation instead of inhibition^45^.

From our in vitro analyses, we propose that DDR targeting through PARP and ATR inhibition and their combination should be further evaluated for a potential clinical use in HPV-negative HNSCC. Pitfalls may include the enhanced cytotoxicity of proliferating mucosal cells in the radiation field and maybe a reliance of effectiveness on the fraction of actively proliferating tumor cells, as both compounds may have especially strong effects in replicating cells. These questions should be addressed in further studies.

## Material and methods

### Cells and cell culture

All cell lines were grown in RPMI (Sigma-Aldrich) supplemented with 10% fetal bovine serum (FBS) (Bio&Sell), 1% Penicillin/Streptomycin (Sigma) at 37 °C, 5% CO_2_ and 100% humidification. Experiments were performed with established HPV-negative HNSCC cell lines HSC4, SAS, UT-SCC-60A and SAT. UT-SCC-60A were obtained as a kind gift from Reidar Grénman (University of Turku, Turku, Finland). HSC4, SAS and SAT were obtained as a kind gift from Wolfgang Eicheler (Oncoray, Dresden University of Technology, Dresden, Germany). Cell lines have been available in the laboratory for many years, were described previously^18,19,46^ and basic characteristics are listed in supplementary table 1. HSC4 and SAS are also commercially available (AcceGen -#ABC-TC0420 & #ABL-TC0611, as of 05.01.2026). Tumor cell line identity was validated by a short tandem repeat multiplex assay (Applied Biosystems). IAP inhibition was performed using 1 µM xevinapant (MedChemExpress) and ATR inhibition was performed using 30 nM tuvusertib (MedChemExpress) unless indicated otherwise. PARP inhibition was performed using 1 µM olaparib (MyBiosource).

### Cell proliferation

For cell proliferation analysis, cells were seeded into T25 cell culture flasks and after 4 h treated with substances as indicated. The numbers of resulting cells were assessed after 5 days using a Coulter counter (Beckmann-Coulter).

### X-irradiation

Cells were irradiated at room temperature with 200 kV X-rays (Gulmay RS225, Gulmay Medical Ltd.; 200 kV, 15 mA,0.8 mm Be + 0.5 mm Cu filtering; dose rate of 1.2 Gy/min).

### Cell cycle assessment

Cells were harvested using trypsin, fixed with 70% ethanol, briefly washed with PBS/0.5% BSA/0.1% Triton X-100 and subsequently incubated with PBS/1% BSA/0.2% Triton X-100/DAPI (4′,6-diamidin-2-phenylindol, 1 µg/ml) for 30 min at room temperature in the dark. Cells were washed once with PBS/0.5% BSA/0.1% Triton X-100 before flow cytometric analysis using a MACSQuant10 with MACSQuantify Software (Miltenyi Biotec). The proportion of cells in the respective cell cycle phases was calculated using ModFit LT™ software (Verity Software House, Inc.).

### Flow cytometric assessment of cell death

Flow cytometric measurement of early apoptosis and lytic cell death was performed on a MACSQuant10 with MACSQuantify and Flowlogic Software (Miltenyi Biotec & Inivai) using combined annexin V / DAPI staining. In brief, cells were harvested using full growth medium after detachment using trypsin. After centrifugation (300 g, 5 min) the supernatant was carefully aspirated, the cells were washed once in PBS and after another centrifugation resuspended in annexin V (Miltenyi Biotec)/DAPI (1 mg/ml) staining solution and incubated for 20 min at room temperature in the dark. Afterwards, the cells were placed on ice in the dark for up to 1 h until flow cytometric analysis.

### Colony formation assay

Radiosensitization was determined using preplating colony formation assays. Exponentially growing cells were seeded in low, defined numbers into 6-well cell culture wells (triplicates per condition). Three hours after seeding the cells were treated with inhibitors/cisplatin or solvent control and after further 2 h irradiated. At 24 h after irradiation, the medium was exchanged with medium lacking the substances, except for xevinapant, which was re-added for 6 further days (total of 1 week). Incubation time until colony formation varied between cell lines from 9 to 21 days for non-irradiated samples; irradiated samples were allowed to grow for an extended period of time when colony formation was apparently delayed. The number of colonies containing more than 50 cells was assessed. Single experiments always contained the full set of substances and radiation doses as indicated.

### Data evaluation

Data analysis was performed using Excel (Microsoft) and GraphPad Prism 6 (GraphPad Software). Statistical differences were generally evaluated using a paired two-tailed Student’s t-test.

## Supplementary Information

Below is the link to the electronic supplementary material.

Supplementary Material 1

## Data Availability

All data generated or analysed during this study are included in this published article [and its supplementary information files].

## References

[1] Ahmadi, N., Chan, M., Huo, Y. R., Sritharan, N. & Chin, R. Y. Survival outcome of tonsillar squamous cell carcinoma (TSCC) in the context of human papillomavirus (HPV): A systematic review and meta-analysis. *Surgeon* **17**, 6–14. 10.1016/j.surge.2018.04.009 (2019). 29843958 10.1016/j.surge.2018.04.009

[2] Avril, D., Foy, J. P., Bouaoud, J., Gregoire, V. & Saintigny, P. Biomarkers of radioresistance in head and neck squamous cell carcinomas. *Int. J. Radiat. Biol.* **99**, 583–593. 10.1080/09553002.2022.2110301 (2023). 35930497 10.1080/09553002.2022.2110301

[3] Johnson, D. E. et al. Head and neck squamous cell carcinoma. *Nat. Rev. Dis. Primers* **6**, 92. 10.1038/s41572-020-00224-3 (2020). 33243986 10.1038/s41572-020-00224-3 PMC7944998

[4] Krishnamurthy, S. et al. The dogma of Cetuximab and Radiotherapy in head and neck cancer—A dawn to dusk journey. *Clin. Transl. Radiat. Oncol.* **34**, 75–81. 10.1016/j.ctro.2022.03.009 (2022). 35356388 10.1016/j.ctro.2022.03.009 PMC8958314

[5] Matzinger, O. et al. The radiosensitizing activity of the SMAC-mimetic, Debio 1143, is TNFalpha-mediated in head and neck squamous cell carcinoma. *Radiother. Oncol.* **116**, 495–503. 10.1016/j.radonc.2015.05.017 (2015). 26096848 10.1016/j.radonc.2015.05.017

[6] Tao, Y. et al. Extended follow-up of a phase 2 trial of xevinapant plus chemoradiotherapy in high-risk locally advanced squamous cell carcinoma of the head and neck: a randomised clinical trial. *Eur. J. Cancer* **183**, 24–37. 10.1016/j.ejca.2022.12.015 (2023). 36796234 10.1016/j.ejca.2022.12.015

[7] Bourhis, J. et al. Xevinapant or placebo plus platinum-based chemoradiotherapy in unresected locally advanced squamous cell carcinoma of the head and neck (TrilynX): A randomized phase III study. *J. Clin. Oncol.*10.1200/JCO-25-00272 (2025). 40902136 10.1200/JCO-25-00272 PMC12509437

[8] Sobti, A., Skinner, H. & Wilke, C. T. Predictors of radiation resistance and novel radiation sensitizers in head and neck cancers: Advancing radiotherapy efficacy. *Semin. Radiat. Oncol.* **35**, 224–242. 10.1016/j.semradonc.2025.02.008 (2025). 40090749 10.1016/j.semradonc.2025.02.008

[9] Viktorsson, K. et al. Advances in molecular targeted therapies to increase efficacy of (chemo)radiation therapy. *Strahlenther. Onkol.* **199**, 1091–1109. 10.1007/s00066-023-02064-y (2023). 37041372 10.1007/s00066-023-02064-y PMC10673805

[10] Guo, S. et al. Research progress of ATR small molecule inhibitors in cancer therapy. *Eur. J. Med. Chem.* **296**, 117804. 10.1016/j.ejmech.2025.117804 (2025). 40479893 10.1016/j.ejmech.2025.117804

[11] Ngoi, N. Y. L. et al. Targeting ATR in patients with cancer. *Nat. Rev. Clin. Oncol.* **21**, 278–293. 10.1038/s41571-024-00863-5 (2024). 38378898 10.1038/s41571-024-00863-5

[12] Jo, U. et al. The novel ATR inhibitor M1774 induces replication protein overexpression and broad synergy with DNA-targeted anticancer drugs. *Mol. Cancer Ther.* **23**, 911–923. 10.1158/1535-7163.MCT-23-0402 (2024). 38466804 10.1158/1535-7163.MCT-23-0402 PMC11555614

[13] Hanna, C. et al. Pharmacokinetics, safety, and tolerability of olaparib and temozolomide for recurrent glioblastoma: Results of the phase I OPARATIC trial. *Neuro Oncol.* **22**, 1840–1850. 10.1093/neuonc/noaa104 (2020). 32347934 10.1093/neuonc/noaa104 PMC7746945

[14] Le Tourneau, C. et al. Phase I trial of Debio 1143, an antagonist of inhibitor of apoptosis proteins, combined with cisplatin chemoradiotherapy in patients with locally advanced squamous cell carcinoma of the head and neck. *Clin. Cancer Res.* **26**, 6429–6436. 10.1158/1078-0432.CCR-20-0425 (2020). 32994295 10.1158/1078-0432.CCR-20-0425

[15] Stanislawiak-Rudowicz, J. et al. The use of Ctrough for the therapeutic drug monitoring of olaparib in patients with ovarian cancer. *Eur. Rev. Med. Pharmacol. Sci.* **26**, 9426–9436. 10.26355/eurrev_202212_30694 (2022). 36591851 10.26355/eurrev_202212_30694

[16] Xiao, R. et al. Dual antagonist of cIAP/XIAP ASTX660 sensitizes HPV(-) and HPV(+) head and neck cancers to TNFalpha, TRAIL, and radiation therapy. *Clin. Cancer Res.* **25**, 6463–6474. 10.1158/1078-0432.CCR-18-3802 (2019). 31266830 10.1158/1078-0432.CCR-18-3802 PMC6825532

[17] Uzunparmak, B. et al. Caspase-8 loss radiosensitizes head and neck squamous cell carcinoma to SMAC mimetic-induced necroptosis. *JCI Insight*10.1172/jci.insight.139837 (2020). 33108350 10.1172/jci.insight.139837 PMC7714407

[18] Kasten-Pisula, U. et al. Cellular and tumor radiosensitivity is correlated to epidermal growth factor receptor protein expression level in tumors without EGFR amplification. *Int. J. Radiat. Oncol. Biol. Phys.* **80**, 1181–1188. 10.1016/j.ijrobp.2011.02.043 (2011). 21514063 10.1016/j.ijrobp.2011.02.043

[19] Oetting, A. et al. Impaired DNA double-strand break repair and effective radiosensitization of HPV-negative HNSCC cell lines through combined inhibition of PARP and Wee1. *Clin. Transl. Radiat. Oncol.* **41**, 100630. 10.1016/j.ctro.2023.100630 (2023). 37180052 10.1016/j.ctro.2023.100630 PMC10172863

[20] Busch, C. J. et al. Similar cisplatin sensitivity of HPV-positive and -negative HNSCC cell lines. *Oncotarget* **7**, 35832–35842. 10.18632/oncotarget.9028 (2016). 27127883 10.18632/oncotarget.9028 PMC5094966

[21] Lacas, B. et al. Meta-analysis of chemotherapy in head and neck cancer (MACH-NC): An update on 107 randomized trials and 19,805 patients, on behalf of MACH-NC group. *Radiother. Oncol.* **156**, 281–293. 10.1016/j.radonc.2021.01.013 (2021). 33515668 10.1016/j.radonc.2021.01.013 PMC8386522

[22] Ahmed, S. U. et al. Selective inhibition of parallel DNA damage response pathways optimizes radiosensitization of glioblastoma stem-like cells. *Cancer Res.* **75**, 4416–4428. 10.1158/0008-5472.CAN-14-3790 (2015). 26282173 10.1158/0008-5472.CAN-14-3790

[23] Karnak, D. et al. Combined inhibition of Wee1 and PARP1/2 for radiosensitization in pancreatic cancer. *Clin. Cancer Res.* **20**, 5085–5096. 10.1158/1078-0432.CCR-14-1038 (2014). 25117293 10.1158/1078-0432.CCR-14-1038 PMC4184968

[24] Parsels, L. A. et al. PARP1 trapping and DNA replication stress enhance radiosensitization with combined WEE1 and PARP inhibitors. *Mol. Cancer Res.* **16**, 222–232. 10.1158/1541-7786.MCR-17-0455 (2018). 29133592 10.1158/1541-7786.MCR-17-0455 PMC5805596

[25] Guster, J. D. et al. The inhibition of PARP but not EGFR results in the radiosensitization of HPV/p16-positive HNSCC cell lines. *Radiother. Oncol.* **113**, 345–351. 10.1016/j.radonc.2014.10.011 (2014). 25467050 10.1016/j.radonc.2014.10.011

[26] Vance, S. et al. Selective radiosensitization of p53 mutant pancreatic cancer cells by combined inhibition of Chk1 and PARP1. *Cell Cycle* **10**, 4321–4329. 10.4161/cc.10.24.18661 (2011). 22134241 10.4161/cc.10.24.18661 PMC3272262

[27] Hintelmann, K. et al. Dual inhibition of PARP and the intra-S/G2 cell cycle checkpoints results in highly effective radiosensitization of HPV-positive hnscc cells. *Front Oncol* **11**, 683688. 10.3389/fonc.2021.683688 (2021). 34354944 10.3389/fonc.2021.683688 PMC8329549

[28] Parsels, L. A. et al. Combinatorial efficacy of olaparib with radiation and ATR inhibitor requires PARP1 protein in homologous recombination-proficient pancreatic cancer. *Mol. Cancer Ther.* **20**, 263–273. 10.1158/1535-7163.MCT-20-0365 (2021). 33268569 10.1158/1535-7163.MCT-20-0365 PMC7867626

[29] Kristeleit, R. et al. DDRiver EOC 302: A Randomised Phase 2 Study Of Tuvusertib With Niraparib Or Lartesertib In Patients With Epithelial OvarianCancer That Has Progressed On Prior PARP Inhibitor Therapy. *International Journal of Gynecological Cancer*, **35** (2), 101597.10.1016/j.ijgc.2024.101597

[30] Yap, T.A. et al. A phase I study of highly potent oral ATR inhibitor (ATRi) tuvusertib plus oral PARP inhibitor (PARPi) niraparib in patients with solidtumors. *J Clin Oncol ***42**, 3018 (2024). 10.1200/JCO.2024.42.16_suppl.3018.

[31] Karam, S. D. et al. Final report of a phase I trial of olaparib with cetuximab and radiation for heavy smoker patients with locally advanced head and neck cancer. *Clin. Cancer Res.* **24**, 4949–4959. 10.1158/1078-0432.CCR-18-0467 (2018). 30084837 10.1158/1078-0432.CCR-18-0467 PMC6873707

[32] Navran, A. et al. Phase I feasibility study of olaparib in combination with loco-regional radiotherapy in head and neck squamous cell carcinoma. *Clin. Transl. Radiat. Oncol.* **44**, 100698. 10.1016/j.ctro.2023.100698 (2024). 38021094 10.1016/j.ctro.2023.100698 PMC10654000

[33] Sheikh, H., Ryder, D., Bateman, A., Chalmers, A. & Jackson, A. Radiotherapy and olaparib in combination for carcinoma of the oesophagus: A phase I study. *Clin. Transl. Radiat. Oncol.* **40**, 100614. 10.1016/j.ctro.2023.100614 (2023). 36949958 10.1016/j.ctro.2023.100614 PMC10025123

[34] de Haan, R. et al. Phase I and pharmacologic study of olaparib in combination with high-dose radiotherapy with and without concurrent cisplatin for non-small cell lung cancer. *Clin. Cancer Res.* **27**, 1256–1266. 10.1158/1078-0432.CCR-20-2551 (2021). 33262140 10.1158/1078-0432.CCR-20-2551

[35] Derby, S. et al. Concurrent olaparib and radiation therapy in older patients with newly diagnosed glioblastoma: The phase 1 dose-escalation PARADIGM trial. *Int. J. Radiat. Oncol. Biol. Phys.* **118**, 1371–1378. 10.1016/j.ijrobp.2024.01.011 (2024). 38211641 10.1016/j.ijrobp.2024.01.011

[36] Bhamidipati, D., Haro-Silerio, J. I., Yap, T. A. & Ngoi, N. PARP inhibitors: Enhancing efficacy through rational combinations. *Br J Cancer* **129**, 904–916. 10.1038/s41416-023-02326-7 (2023). 37430137 10.1038/s41416-023-02326-7 PMC10491787

[37] Uppaluri, R. et al. Neoadjuvant and adjuvant pembrolizumab in locally advanced head and neck cancer. *N. Engl. J. Med.* **393**, 37–50. 10.1056/NEJMoa2415434 (2025). 40532178 10.1056/NEJMoa2415434

[38] Machiels, J. P. et al. Pembrolizumab plus concurrent chemoradiotherapy versus placebo plus concurrent chemoradiotherapy in patients with locally advanced squamous cell carcinoma of the head and neck (KEYNOTE-412): A randomised, double-blind, phase 3 trial. *Lancet Oncol.* **25**, 572–587. 10.1016/S1470-2045(24)00100-1 (2024). 38561010 10.1016/S1470-2045(24)00100-1

[39] Tao, Y. et al. Pembrolizumab versus cetuximab concurrent with radiotherapy in patients with locally advanced squamous cell carcinoma of head and neck unfit for cisplatin (GORTEC 2015–01 PembroRad): A multicenter, randomized, phase II trial. *Ann. Oncol.* **34**, 101–110. 10.1016/j.annonc.2022.10.006 (2023). 36522816 10.1016/j.annonc.2022.10.006

[40] Lee, N. Y. et al. Avelumab plus standard-of-care chemoradiotherapy versus chemoradiotherapy alone in patients with locally advanced squamous cell carcinoma of the head and neck: A randomised, double-blind, placebo-controlled, multicentre, phase 3 trial. *Lancet Oncol.* **22**, 450–462. 10.1016/S1470-2045(20)30737-3 (2021). 33794205 10.1016/S1470-2045(20)30737-3

[41] Bourhis, J. et al. LBA35 Avelumab-cetuximab-radiotherapy versus standards of care in patients with locally advanced squamous cell carcinoma of head andneck (LA-SCCHN): Randomized phase III GORTEC-REACH trial. *Annals of oncology, 2021,* **32**(Suppl 5), S1310 (2021).10.1016/j.annonc.2021.08.2112

[42] Gillison, M. L. et al. Radiotherapy plus cetuximab or cisplatin in human papillomavirus-positive oropharyngeal cancer (NRG Oncology RTOG 1016): A randomised, multicentre, non-inferiority trial. *Lancet* **393**, 40–50. 10.1016/S0140-6736(18)32779-X (2019). 30449625 10.1016/S0140-6736(18)32779-X PMC6541928

[43] Mehanna, H. et al. Radiotherapy plus cisplatin or cetuximab in low-risk human papillomavirus-positive oropharyngeal cancer (De-ESCALaTE HPV): An open-label randomised controlled phase 3 trial. *Lancet* **393**, 51–60. 10.1016/S0140-6736(18)32752-1 (2019). 30449623 10.1016/S0140-6736(18)32752-1 PMC6319250

[44] Rischin, D. et al. Randomized trial of radiation therapy with weekly cisplatin or cetuximab in low-risk hpv-associated oropharyngeal cancer (TROG 12.01)—A trans-tasman radiation oncology group study. *Int. J. Radiat. Oncol. Biol. Phys.* **111**, 876–886. 10.1016/j.ijrobp.2021.04.015 (2021). 34098030 10.1016/j.ijrobp.2021.04.015

[45] Alsahafi, E. N. et al. EGFR overexpression increases radiotherapy response in HPV-positive head and neck cancer through inhibition of DNA damage repair and HPV E6 downregulation. *Cancer Lett.* **498**, 80–97. 10.1016/j.canlet.2020.10.035 (2021). 33137407 10.1016/j.canlet.2020.10.035

[46] Bussmann, L. et al. Analyzing tyrosine kinase activity in head and neck cancer by functional kinomics: Identification of hyperactivated Src family kinases as prognostic markers and potential targets. *Int. J. Cancer* **149**, 1166–1180. 10.1002/ijc.33606 (2021). 33890294 10.1002/ijc.33606

