# Supplementary material for: Comparing and combining xevinapant with ATR and PARP inhibition for the radiosensitization of HPV-negative HNSCC cells
Source: Sci Rep. 2026 Feb 11;16:5882. doi: 10.1038/s41598-026-38550-3 (PMC12894864; doi:10.1038/s41598-026-38550-3)
Supplement: Supplementary file 1 — Supplementary Material 1 [file 41598_2026_38550_MOESM1_ESM.pdf]

Comparing and combining xevinapant with ATR and PARP inhibition for the radiosensitization of HPV-negative HNSCC cells

Supplementary Information

Cell line	Sex	Location	Selected mutations
HSC4	male	tongue	PolQ Val310Gly ; TP53 p.Arg248Gln ; TERT c.1-124C>T ; CDKN2A p.Arg80* ; PIK3CA p.Glu545Lys ; PTEN pCys136Trp
UT-SCC-60A	male	tonsil/oral cavity	TP53 p.Arg342* ; KMT2D p.P1465L & p.P1465S ; PDE4DIP p.R8H ; BRIP1 p.G690R
SAS	female	tongue	CASP8 p.Ser397Phe ; TP53 p.Glu336* ; SDHA p.Leu649fs*4 ; SETD1B p.Ser1400fs*18 ; EPHA2 p.Leu551fs*38
SAT	n.a.	HNSCC	BRCA1 p.Met1649Thr ; TP53 p.Arg110Leu & p.Glu271* ; SDHA p.Leu649fs*4 ; ASXL p.Gly738*
FaDu	male	hypopharynx	TP53 p.Arg248Leu & c.673-1G>A (slice site) ; CDKN2A c.151-1G>T (splice site) ; FAT1 p.Lys3277Asnfs*4 ; VHL p.M1I

Supplementary Table 1. Utilized cell lines. *Mutation data were obtained from publicly accessible databases https://www.cellosaurus.org/index.html and https://cellmodelpassports.sanger.ac.uk/ on 18.11.2025 and for UT-SCC-60A, which was not listed in the cellmodelspassports database, additionally from Lephikova et al. (DOI: 10.1158/1535-7163.MCT-17-0733). Mutations in caspase 8 could be highly relevant for the response towards SMAC mimetics (DOI: 10.1172/jci.insight.139837) and in PolQ and BRCA1 for the response towards PARP inhibition, but the listed missense mutations have not been functionally characterized. "p." refers to protein level changes, "c." to DNA level changes. "*" = premature termination.*

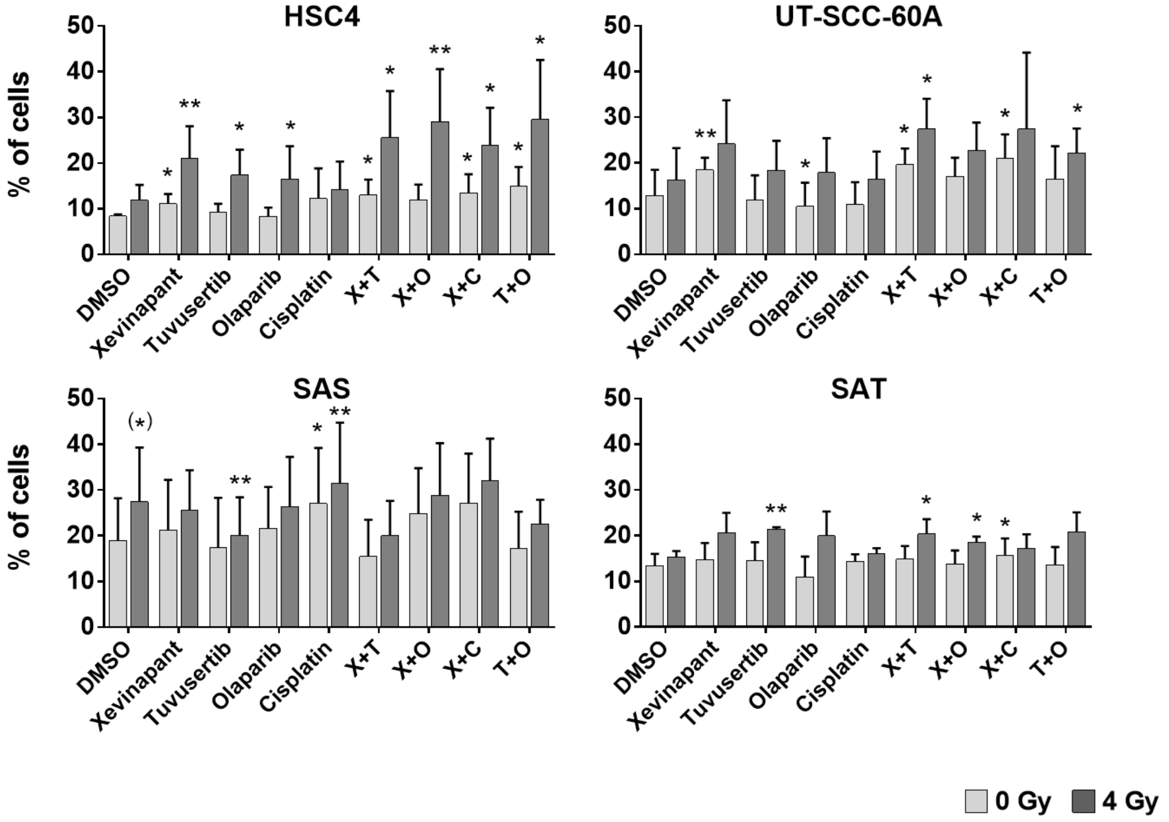

Supplementary Figure 1. Cell death induction. *Total cell death numbers as obtained as the sum of early apoptosis and lytic cell death from the experiments presented in Figure 3. Significant differences to the respective irradiated or unirradiated DMSO control are indicated with *, ** and *** indicating p<0.05, p<0.01 and p<0.001, respectively (paired, two-tailed Student's t-test). Asterisk in brackets indicates a significant difference between irradiated and non-irradiated solvent controls. Results are based on at least 4 individual experiments per cell line.*

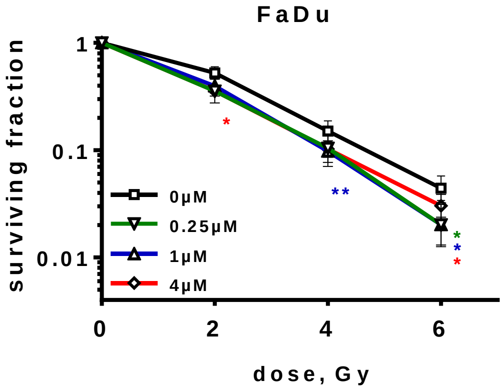

Supplementary Figure 2. Radiosensitization of FaDu cells through xevinapant. *Cells were seeded in defined low numbers and after 3 hours treated with xevinapant. Two hours later the cells were irradiated. Medium was exchanged to medium without xevinapant after 1 week and cultures were incubated until formation of colonies. Significant differences to the respective solvent controls are indicated with *, ** and *** indicating $p<0.05$, $p<0.01$ and $p<0.001$, respectively (paired, two-tailed Student's t-test). Results are based on 4 individual experiments. Dose-response curves display mean and standard deviation.*
